# A bibliometric analysis of immunotherapy for atherosclerosis: trends and hotspots prediction

**DOI:** 10.3389/fimmu.2024.1493250

**Published:** 2024-11-19

**Authors:** Jing-Hui Wang, Guan-Rui Pan, Long Jiang

**Affiliations:** ^1^ Department of Cardiovascular Medicine, The Second Affiliated Hospital of Nanchang University, Nanchang, Jiangxi, China; ^2^ Nanchang University Queen Mary School, Nanchang, Jiangxi, China

**Keywords:** atherosclerosis, immunotherapy, bibliometrics, hotspots, trends, visualization

## Abstract

**Introduction:**

An increasing number of studies have demonstrated that immunotherapy may play a significant role in treating Atherosclerosis and has emerged as a promising therapy in this field. The aim of this study is to provide a comprehensive perspective through bibliometric analysis and investigate the existing hotspots and frontiers.

**Methods:**

This study searched records from Web of Science, PubMed, and Scopus from January 1, 1999, to May 27, 2023. By using bibliometric software CiteSpace (6.3.R1) and VOSviewer (1.6.19), co-occurrence analysis was used to count the frequency of co-occurrence of certain elements (e.g., countries, regions, institutions, etc.), cluster analysis was used to classify keywords, and burst analysis was used to identify research trends and hotspots.

**Results:**

The results showed that the number of annual publications has grown in a fluctuating manner; the USA, China, and the Netherlands have the highest numbers of publications, and the top three institutions are located in the Netherlands, Sweden, and the USA. In addition, Nilsson J published the highest number of papers; Ridker PM and his article “Anti-inflammatory Therapy with Canakinumab for Atherosclerotic Disease” have played prominent roles. The top four Journals with the highest numbers of publications are “Arteriosclerosis Thrombosis and Vascular Biology”, “Frontiers in Cardiovascular Medicine”, “Circulation” and “Vaccine”. In addition, keyword analysis indicates that inflammation, nanoparticles, adverse events associated with immune checkpoint inhibitors, T cells and tumor necrosis factor will be future research hotspots.

**Discussion:**

This study provides a comprehensive bibliometric analysis of immunotherapy in atherosclerosis, offering insights that advance scientific understanding. It not only assists researchers in grasping the current hotspots in this field but also reveals potential directions for future investigation. Moreover, future studies can optimize immunotherapy strategies based on hotspot predictions to decelerate the progression of atherosclerosis.

## Introduction

Atherosclerosis is the major underlying cause of atherosclerotic cardiovascular disease (ASCVD) (1, 2). The population susceptible to ASCVD has expanded to include younger individuals and women, leading to a global mortality rate of 17.9 million per year and making ASCVD the leading cause of death (3). The widespread use of statins contributes to a reduction in low-density lipoprotein cholesterol (LDL-C) and lowers the relative risk of all-cause death by 8%, establishing statins as the cornerstone of ASCVD treatment (4). In addition to statins, various lipid-lowering therapies, including proprotein convertase subtilisin/kexin type-9 (PCSK9) inhibitors, have been developed. These novel drugs can significantly decrease LDL-C levels from 14% to 75% (5), and they can reduce major adverse cardiovascular events (MACE) by 13% to 30% (6–8). Although these lipid-lowering strategies can reduce LDL-C even to levels under 70 mg/dl, there remains a persistent residual cardiovascular risk (54%-61%), suggesting that the current lipid-lowering therapies are still ineffective (9). Therefore, additional research and exploration of new alternative therapeutic methods are needed.

Recently, immune factors have been found to play pivotal roles in AS (10). In recent decades, a number of studies have indicated that atherosclerosis is strongly associated with the immune system and inflammation (11, 12). Therefore, an increasing number of researchers are paying attention to the use of immunotherapy for AS (13). Clinical trials have shown the effectiveness of IL-1β and IL-6 inhibitors, specifically the Canakinumab and Ziltivekimab, in reducing cardiovascular events. In a clinical study involving 10,061 participants, the IL-1β antagonist canakinumab significantly decreased the incidence of recurrent cardiovascular events by 15%. However, patients receiving Canakinumab experienced side effects such as neutropenia and thrombocytopenia, and there was a notable increase in deaths due to infections and sepsis (14). Another study focused one IL-6 inhibitor, ziltivekimab, which has been demonstrated to be effective in reducing inflammatory and thrombotic biomarkers in patients over 18 years of age with chronic stage 3-5 kidney disease or high-sensitivity C-reactive protein (CRP) levels greater than 2 mg/L, without significant adverse effects (15). Colchicine also exhibits anti-inflammatory properties in patients post-myocardial infarction and may reduce the risk of ischemic heart disease (IHD). One study involving 4,745 participants indicated that colchicine exerted an anti-inflammatory effect in patients with myocardial infarction and effectively reduced the risk of IHD compared with placebo (16). Although the experimental group frequently experienced adverse reactions such as nausea, vomiting and pneumonia, which is a serious adverse event, the overall incidence of adverse reactions did not differ significantly from that of the placebo group (16). Despite the gradual increase of related research, these studies have yet to be systematically analyzed.

Bibliometrics is a quantitative science that can identify influential articles by evaluating the influence and impact of research, and it has had a considerable impact on medical research (17, 18). CiteSpace and VOSviewer are two commonly used bibliometric analysis software programs that can provide concise scientific information and practical guidance (19). There has yet to be a bibliometric analysis in this area. This absence presents a critical gap in the literature, leaving researchers lack a comprehensive understanding of current trends, key articles, influential authors and institutions, collaboration networks, and research hotspots, which are vital for advancing in this field. Therefore, this study aimed to perform a bibliometric analysis utilizing CiteSpace and VOSviewer to (1) explore the research status, (2) visualize collaboration networks between regions, institutions and authors, (3) identify present hotspots and typical articles, (4) direct interest in the future, and (5) provide support for researchers and facilitate the advancement of this field.

## Materials and methods

### Data sources

We collected literature for our study from three databases: the Web of Science Core Collection (WoSCC), Scopus, and PubMed. All the searches were conducted on the same day to avoid potential bias due to daily database updates. The WoSCC, Scopus, and PubMed databases were three main databases used in medical field. WoSCC and Scopus are two comprehensive literature database covering multi-disciplinary fields enables researchers to obtain comprehensive literature support. And PubMed is a database specializes in biomedical literature, providing access to a wide collection of articles that may not be fully included in WoSCC or Scopus. These three were selected to ensure that data sources for bibliometric research are broad, authoritative, and diverse, providing the quality of bibliographic data to support comprehensive bibliometric analyses.

### Time span

Our search spanned from January 1, 1999, to May 27, 2023.

### Inclusion criteria

We included articles and reviews published in English related to the use of immunotherapy for atherosclerosis.

### Exclusion criteria

This study excluded articles not published in the English language. Moreover, publications that were not original articles or reviews were also excluded.

### Search strategy

Taking WoS as an example, the search session queries were: TS=(Arteriosclerosis OR Arterioscleroses OR Fibroatheroma OR Fibroatheromas OR Arterial Fatty Streak OR Arterial Fatty Streaks OR Atherosclerotic Plaques OR Atherosclerotic Plaque OR Atheroma OR Atheromas OR Atheromatous Plaques OR Atheromatous Plaque OR Atherosclerosis OR Fibroatheromatous Plaques OR Fibroatheromatous Plaque) AND TS=(Immunotherapy OR Immunotherapies OR immunotherapeutic OR Immune Checkpoint Inhibitors OR PD-1 OR anti-PD-1/L1 OR CTLA-4 OR nivolumab OR pembrolizumab OR lambrolizumab OR avelumab OR ipilimumab OR cemiplimab OR atezolizumab OR durvalumab OR anti-CTLA-4 OR tremelimumab OR ticilimumab OR utomilumab). The form of search session queries had some changes in different databases with the same content.

### Data standardization

Before analysis, the synonyms were unified by making “citespace.alias file” and “VOSviewer thesaurus file” in CiteSpace and VOSviewer, respectively, which is necessary to maintain consistency and produce more accurate results. In addition, nonsense words such as “123” and “a” were excluded by making an exclusion file.

### Screening process

The literature screening process comprised multiple stages. Initially, the titles and abstracts of the 1503 retrieved articles were meticulously screened to eliminate irrelevant literature. We subsequently examined the full texts of the selected articles to evaluate their relevance to our research questions. Finally, 174 articles that met the requirements were obtained. The screening and retrieval procedures were independently performed by two authors (Jinghui Wang & Guanrui Pan). In instances of dissent, resolution was diligently sought through rigorous discussions. The results were stored in the download *.txt format (**Supplementary Figure S1**).

### Ethical consent

No ethical support was needed in this study.

### Data analysis and visualization

This study employed bibliometric analysis to examine publications on the use of immunotherapy for atherosclerosis. The tools of choice for this task were advanced CiteSpace (6.3. R1), VOSviewer (1.6.20), and Microsoft Excel 2021. The analysis included various aspects of the publications, including the annual count, geographical distribution, contributing institutions, featured journals, authors, citation frequency, and keywords. The goal was to distinguish the defining characteristics of these papers and present a descriptive overview.

CiteSpace, a Java-based application developed by ChaoMei Chen (20), excels in collating keyword bursts, thereby effectively highlighting emerging trends and evolving shifts in research hotspots (21, 22). It facilitates mining and visualization of knowledge from bibliographic databases, enabling the exploration of authorship, collaborations at the national and institutional levels, knowledge domains, emergent disciplines, and future research trajectories. VOSviewer, a program developed by Eck and Waltman of Leiden University, is specifically designed for constructing scientometric networks and visualizing knowledge graphs (23). It is renowned for its robust visualization capabilities, which can vividly illustrate collaborations between research topics (24–26).

The filtered data were imported into CiteSpace 6.3.R1 as a “download_*.txt” file, with “Data” selected for the removal of duplicates. The time span for each year was set from 1999.1 to 2023.5, and the top 50 authors, institutions, and keywords for each time period were filtered by frequency of occurrence. The parameters of visualization were set as follows: 1) time slicing was set from January 1, 1999, to December 31, 2023, with a time slice interval of n=1; 2) a g-index of k=25 was set, and the top 50 and 10% of the most cited or co-occurring items were selected; g-index provides a more comprehensive measure of impact by combining the number of highly cited papers and the total number of citations; and 3) other parameters were set according to their default conditions. Network pruning was chosen according to the preliminary analyses of pathfinding networks (PFNETs), minimum spanning trees (MSTs), or no network pruning. The link strength and range are set to cosine and intraslice, respectively. Setting it to a cosine value quantifies the similarity between research entities (e.g., keywords, authors, or research organizations), reflecting their proximity in terms of research direction or subject matter through the measurement of angular differences within the vector space. Conversely, setting the link range as an intra-slice value indicates that the study will concentrate on associations among research entities within a defined temporal slice, thereby elucidating research hotspots and collaboration patterns within that specific period. This configuration facilitates the analysis of the evolution of knowledge domains and research trends, enabling researchers to accurately capture and compare the similarities and correlations of research entities within a controlled temporal framework. The selection criteria varied from visualization to visualization due to the scarcity of nodes. VOSviewer (1.6.20) was used to analyze the total link strength and citation counts for each country/region, the citations of cocited journals and authors and the journal distributions of publications by importing “download_*.txt”. Additionally, Microsoft Excel 2021 served as the fundamental tool for charting annual publication trends. The journal impact factor (IF) and Journal Citation Reports (JCR) for 2023 were also obtained from the Web of Science.

In our investigation, CiteSpace 6.3.R1 was used to conduct comprehensive detection and visualization collaboration of countries/regions and institutions, authorship, journals, keywords, and references. Generally, a collaboration network comprises nodes, labels and linages. The size of nodes represents the number of publications from a country, institution or author and the number of times a keyword or reference occurred. Different colored circles are wrapped around each node, indicating the year of publication. Linkages among nodes represent connections, and the thickness reflects the co-occurrence intensity and degree of cooperation. In addition, the outermost purple circle of a node indicates betweenness centrality over 0.1. Betweenness centrality is the number of times a node acts as the shortest bridge for two other nodes; the more times it is a bridge, the greater the degree of centrality. A value over 0.1 signifies a significant node connecting two nodes (27).

With the clustering capacity of CiteSpace, cluster analysis of keywords can separate the elements into various groups according to connectivity and co-occurrence relationships. Each cluster represents the focus of topic selection, and an in-depth discussion of each cluster can yield more valuable insights in this field. A timeline burst map is a method of analysis in which time evolution is added to a cluster map. Modularity Q (Q, Q-score) and the weight mean silhouette S (S-score, value interval [-1,1]) are two indicators used to evaluate the goodness of the clustering map. Q ranges from 0 to 1, and it is used to assess the network structure; Q>0.3 indicates that the structure is significant. S ranges from -1 to 1, and it is a symbol of the level of similarity between cluster members; S>0.5 means the clustering result is rational, and S>0.7 means the result is cogent (28, 29). The keyword clusters were extracted from keywords via CiteSpace using the LLR algorithm. The timeline view can reveal the emergence of new topics or keywords each year and demonstrate the evolution of clusters and period hotspots. This analysis can facilitate longitudinal development in this field and help researchers understand progress in this field more intuitively.

Our study also analyzed burst citations of references and keywords. A burst is an explosion of a word or document in a short time, and burst analysis of cocited references can illustrate a change in research in this field (28, 30). The burst map reveals burst strength, beginning and ending years, and high occurrence periods. In a burst map, the blue line represents the time span, which can be divided into a light line, representing the time before publication, and a dark line, representing the time after publication. The red segment represents the period between the beginning and end of a document’s appearance, i.e., when the reference was cited most frequently. “Year” is the year of publication of the paper, and “Begin” and “End” are the start and end of the burst, respectively. In the burst analysis of reference, we produce the top 25 burst references via CiteSpace, setting the parameter to a minimum Duration=2 and γ=0.8, whereas in the burst analysis of keywords, we produce the top 25 strongest keywords by setting the parameters to a minimum Duration=2 and γ=0.6. Citation bursts can identify fluctuations in keywords, providing crucial insights for predicting future research trends.

Created by more than 10,000 journals from the Web of Science dataset, a dual map overlay can be used in journal analysis and visualization, and it can reflect journal distribution (31). The left side of the map shows the citing journals, and the right side shows the cited journals. The pathways between two groups of journals are shown by colored connected lines, and the circles on the map represent the ratio of authors to the number of publications for each journal, with the length indicating the number of authors and the width indicating the number of publications. Two parameters were used in the dual map: the z score and the f score. The z score represents the cluster fraction of nodes, whereas the f value indicates the number of publications corresponding to a node (32). By introducing these two parameters, we can identify the relationship strength and influence level.

## Results

### Annual publication trend

**Figure 1:**
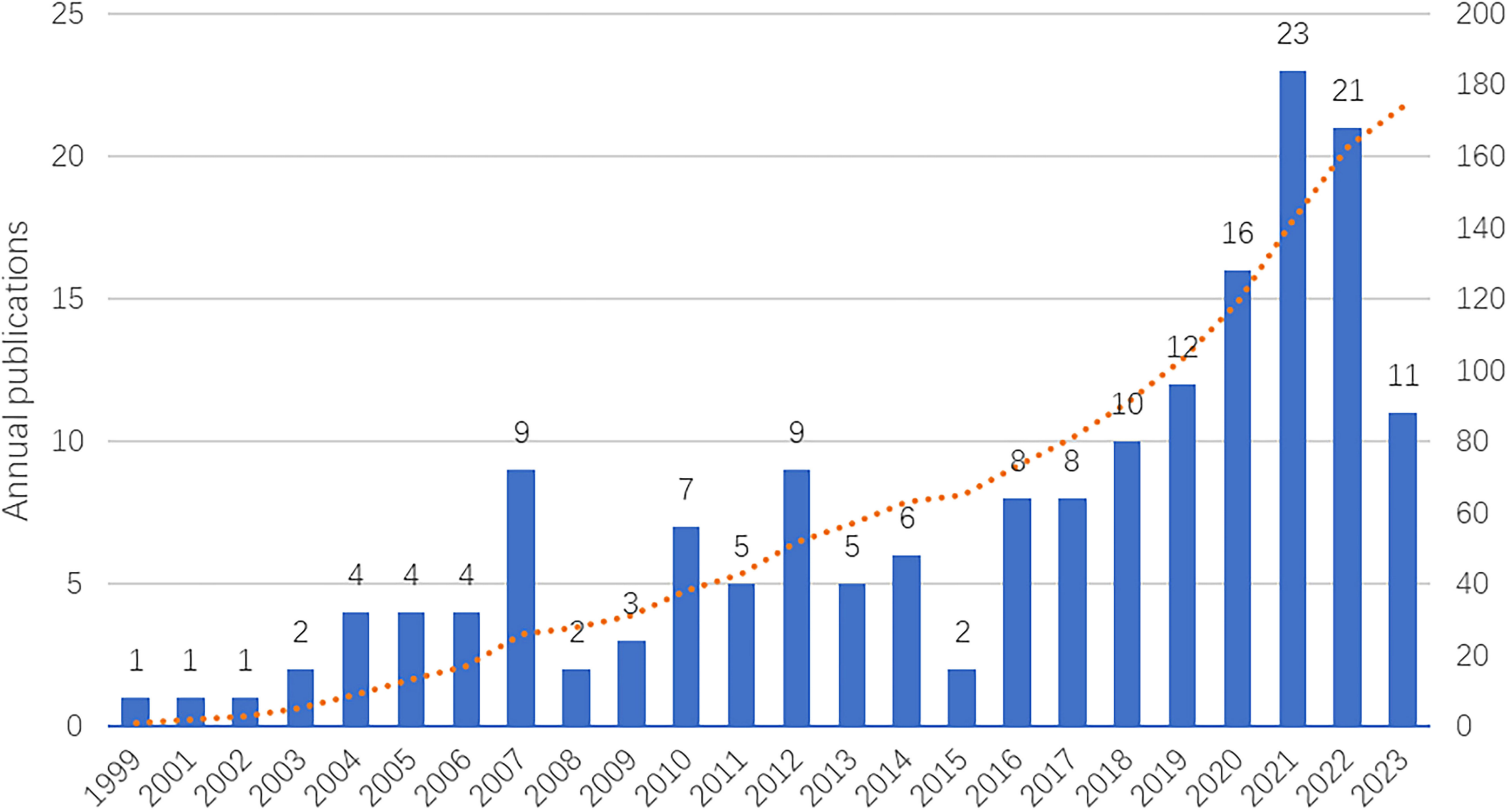
The annual trend of publications and the cumulative publication of immunotherapy of atherosclerosis. The blue bars represent the number of publications of each year, and the red dotted line represents the cumulative publication.

From 1990-2023, 132 articles and 42 reviews were published. The annual publication trend and the cumulative number of publications are shown in **Figure 1**. Overall, the annual publication trend was volatile. The trend can roughly be divided into three stages: 1) from 1999 to 2007, the number of publications grew slowly; 2) from 2008 to 2015, the trend fluctuated substantially; and from 2016 to 2022, the total number of publications grew rapidly, but a decrease was observed in 2022. The highest number of publications (23) was observed at the end of 2021. In addition, the number of publications in 2023 was counted up to May, and the trend cannot be estimated. In general, the exploration of immunotherapy and atherosclerosis was initiated at an early stage, with studies experiencing steady growth in recent years. Nevertheless, the overall research output related to this topic remains relatively limited.

### Country/region and institution co-occurrence

**Figure 2:**
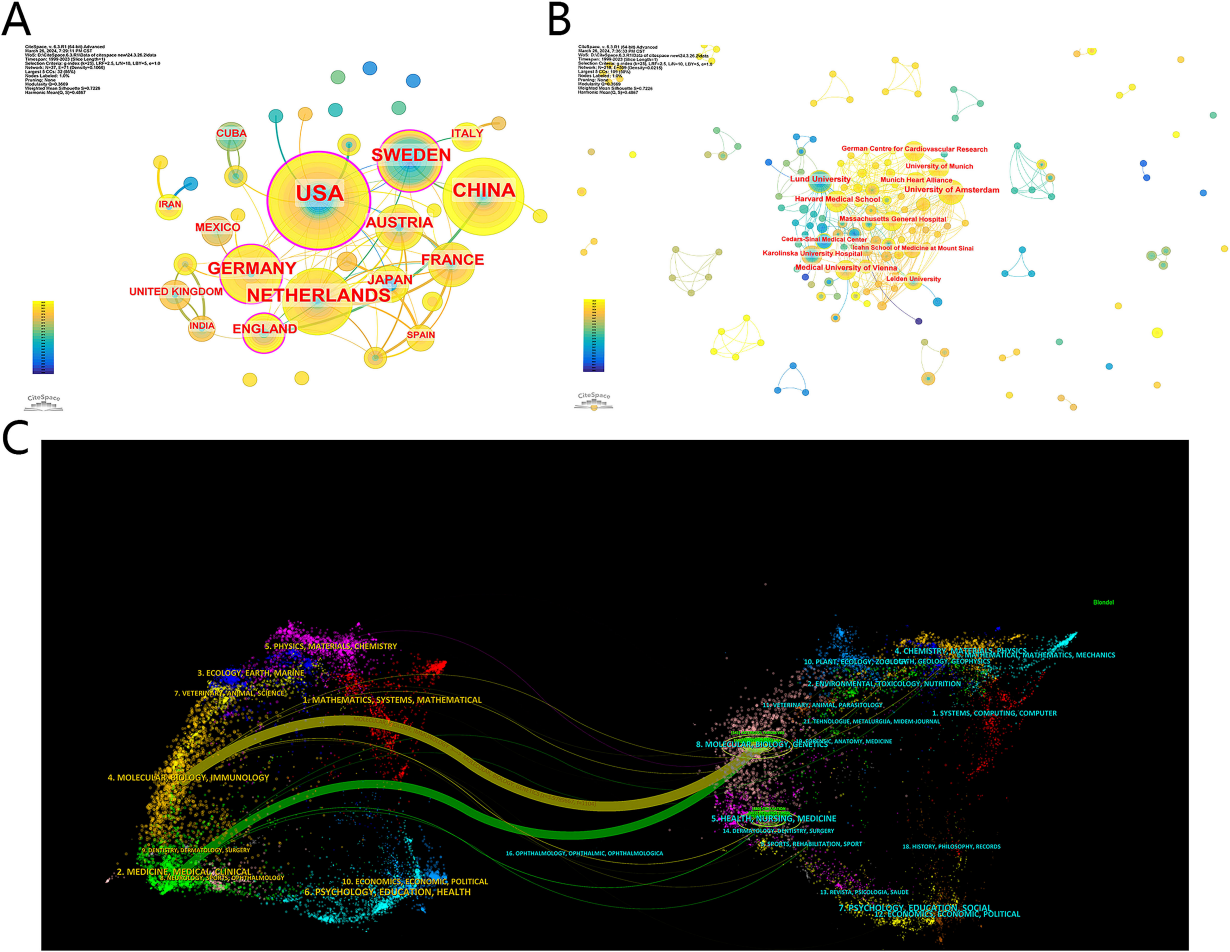
**(A)** Countries/Regions Co-occurrence map in immunotherapy of atherosclerosis. Each node represents a country/region, and different colors of circles represent the year. A node with variable colors means papers are produced in this region in different years. The outmost purple circle means betweenness centrality over 0.1, and only the USA, Sweden, Germany and England got circled in this figure. And collaboration is shown as links between nodes. **(B)** Institutions Co-occurrence map in immunotherapy of atherosclerosis. Each node represents an institution. **(C)** Double image overlay of journals. Parameters: α: 3; citing journal titles (min pubs):10; cited journal titles (min cites): 100; snap to centroids (<Radius): 0. The citing journals are located on the left, and the cited journals are located on the right. Different color clusters represent different fields. The link between two clusters means the collaboration between different research fields.

A total of 218 institutions from 37 countries/regions were included. **Table 1** displays the top 10 countries/regions and institutions. The USA published the highest number of studies (56, 32.2%), followed by China (32, 18.4%), the Netherlands (27, 15.5%), Sweden (23, 13.2%) and Germany (20, 11.5%). In addition, France published the first study in 1999, followed by Sweden (2001) and Japan (2002). In **Figure 2A**, the map has 37 nodes representing each contour/region and 71 connections representing the interactions between regions, with an interaction density of 0.1066. Only 4 countries/regions have a centrality greater than 0.1, including the USA (0.47), Sweden (0.17), Germany (0.17) and England (0.11) (**Figure 2A**), representing the role of the bridge. The total link strength and number of citations were produced by VOSviewer (**Table 1**). The USA has the strongest total link strength (51), followed by the Netherlands (39), Germany (29) and France (25). In terms of total citations, papers from the USA are the most frequently cited, with 1281 citations, followed by those from the Netherlands (1049), Sweden (815), and France (721). Overall, the USA and Europe are the main countries/regions involved in research on the use of immunotherapy for atherosclerosis, and the USA is the most influential, with the most publications, centrality, total link strength and number of citations.

**Table 1:** The top 10 countries/regions and institutions.

Rank	Country/region	Year	N (%)	Centrality	Total link strength	Citations
1	USA	2003	56 (32.18%)	0.47	51	1281
2	China	2006	32 (18.39%)	0.08	6	206
3	Netherlands	2006	27 (15.51%)	0.04	39	1049
4	Sweden	2001	23 (13.22%)	0.17	19	815
5	Germany	2007	20 (11.49%)	0.17	29	579
6	Austria	2007	13 (7.47%)	0.01	13	176
7	France	1999	13 (7.47%)	0.04	25	721
8	Japan	2002	9 (5.17%)	0.00	4	46
9	England	2012	8 (4.60%)	0.11	13	308
10	Italy	2012	6 (3.45%)	0.08	2	119

Centrality: A parameter that assesses the level of connectivity between a node and other nodes within a network. Higher centrality values suggest a more prominent role of the node in the academic domain.

Total Link Strength: A parameter that quantifies the aggregate strength of all connections between a node and other nodes. An increased number of connections and greater strength contribute to a higher total link strength.

The top 10 institutions are located in Germany (3/10), the USA (2/10), Sweden (2/10), the Netherlands (2/10), and Austria (1/10). The University of Amsterdam (15, 8.62%), Lund University (14, 8.05%), Harvard Medical School (12, 6.90%), Medical University of Vienna (12, 6.90%) and University of Munich (9, 5.17%) are the top 5 institutions (**Table 2**). The institution co-occurrence map is shown in **Figure 2B** and contains 218 nodes and 509 connections, with a density of 0.0207. A broad collaboration of institutions is shown with several scattered corporation networks. However, no institution has a centrality over 0.1. Lund University has the greatest centrality of 0.09, followed by the Institute National de la Santé et de la Recherche Médicale (0.05), which is not included in the top 10 institutions.

**Table 2:** Top 10 institutions.

Rank	Institution	Country/region	N (%)	Centrality
1	University of Amsterdam	Netherlands	15 (8.62%)	0.04
2	Lund university	Sweden	14 (8.05%)	0.09
3	Harvard Medical School	USA	12 (6.90%)	0.04
4	Medical University of Vienna	Austria	12 (6.90%)	0.04
5	University of Munich	Germany	9 (5.17%)	0.01
6	Massachusetts General Hospital	USA	9 (5.17%)	0.02
7	Karolinska University Hospital	Sweden	9 (5.17%)	0.04
8	Munich Heart Alliance	Germany	9 (5.17%)	0.01
9	German Centre for Cardiovascular Research	Germany	9 (5.17%)	0.01
10	Leiden University	Netherlands	8 (4.60%)	0.01

Collaboration among countries and institutions is extensive. China, Sweden, the USA, the Netherlands, Germany, and England form the backbone of the map, facilitating collaboration between countries/regions. The USA plays a crucial role as a bridge for collaboration and primarily collaborates with Sweden, the Netherlands, and Germany. With respect to institutional cooperation, Lund University, the University of Amsterdam, Harvard Medical School, and the Medical University of Vienna are important nodes connecting the entire cooperative network.

### Journal and co-cited journals

The top 11 journals were identified by VOSviewer, and the IF and JCR partitions of 2023 were researched online. A total of 174 papers on the use of immunotherapy for atherosclerosis have been published in 116 journals (all journals and their numbers and IFs are shown in **Supplementary Table S1**). Among all the academic journals, 20 journals have an IF greater than 10, and Nature Medicine has the highest IF (58.7) (**Supplementary Table S1**). Most articles and reviews were published in Arteriosclerosis Thrombosis and Vascular Biology (14, 8.05%), followed by Frontiers in Cardiovascular Medicine (6, 3.45%), Circulation (5, 2.87%) and Vaccine (5, 2.87%). Seven journals ranked at Q1 of the JCR are in the top 11 journal list: Arteriosclerosis Thrombosis and Vascular Biology, Circulation, Cardiovascular Research, JACC: CardioOncology, Frontiers in Immunology, Journal of the American College of Cariology (JACC), and European Heart Journal (**Table 3**). More than 1000 cocited journals were extracted via VOSviewer. Arteriosclerosis Thrombosis and Vascular Biology (n=1016) is the most frequently cocited journal, followed by Circulation (803), Circulation Research (406), and Atherosclerosis (406) (**Table 4**). Among the top 10 cocited journals, the New England Journal of Medicine has the highest IF of 96.2, followed by Nature Medicine (58.7) and Circulation (35.5). In addition, with the exception of Atherosclerosis, all the remaining journals are ranked at Q1 in the JCR (**Table 4**).

**Table 3:** Top 11 journal.

Rank	Journal	N(%)	IF (2023)	JCR Partitions (2023)
1	Arteriosclerosis thrombosis and vascular biology	14 (8.05%)	7.4	Q1
2	Frontiers in cardiovascular medicine	6 (3.45%)	2.8	Q2
3	Circulation	5 (2.87%)	35.5	Q1
4	Vaccine	5 (2.87%)	4.5	Q2
5	CARDIOVASCULAR RESEAECH	4 (2.30%)	10.9(2022)	Q1
6	JACC: CardioOncology	4 (2.30%)	12	Q1
7	Frontiers in immunology	4 (2.30%)	5.7	Q1
8	Journal of the American college of cardiology	3 (1.72%)	21.7	Q1
9	European heart journal	3 (1.72%)	37.6	Q1
10	Atherosclerosis	3 (1.72%)	4.9	Q2
11	American journal of physiology	3 (1.72%)	4.1	Q2

**Table 4:** Top 10 cocited journals.

Rank	Journal	Citations	IF (2023)	JCR Partitions (2023)
1	Arteriosclerosis Thrombosis and Vascular Biology	1016	7.4	Q1
2	Circulation	803	35.5	Q1
3	Circulation Research	406	16.5	Q1
4	Atherosclerosis	406	4.9	Q2
5	The New England Journal of Medicine	369	96.2	Q1
6	Journal Of Clinical Investigation	366	13.3	Q1
7	Journal of Immunology	307	3.6	Q2
8	Journal of the American College of Cardiology	264	21.7	Q1
9	Nature Medicine	242	58.7	Q1
10	Journal of Experimental Medicine	241	12.6	Q1

**Figure 2C** shows this dual-map overlay, which effectively illustrates the relationships between journals and their topic positions (33). The left side of the map represents citing journals, whereas the right side of the map represents cited journals. In **Figure 2C**, two main pathways are evident: a yellow and a green pathway. These pathways indicate that the journal of Molecular, Biology, Immunology and Medicine, Medical, Clinical consistently cite articles in the journal of Molecular, Biology, and Genetics, highlighting a clear citation relationship in these fields.

### Authors and co-cited authors

**Figure 3:**
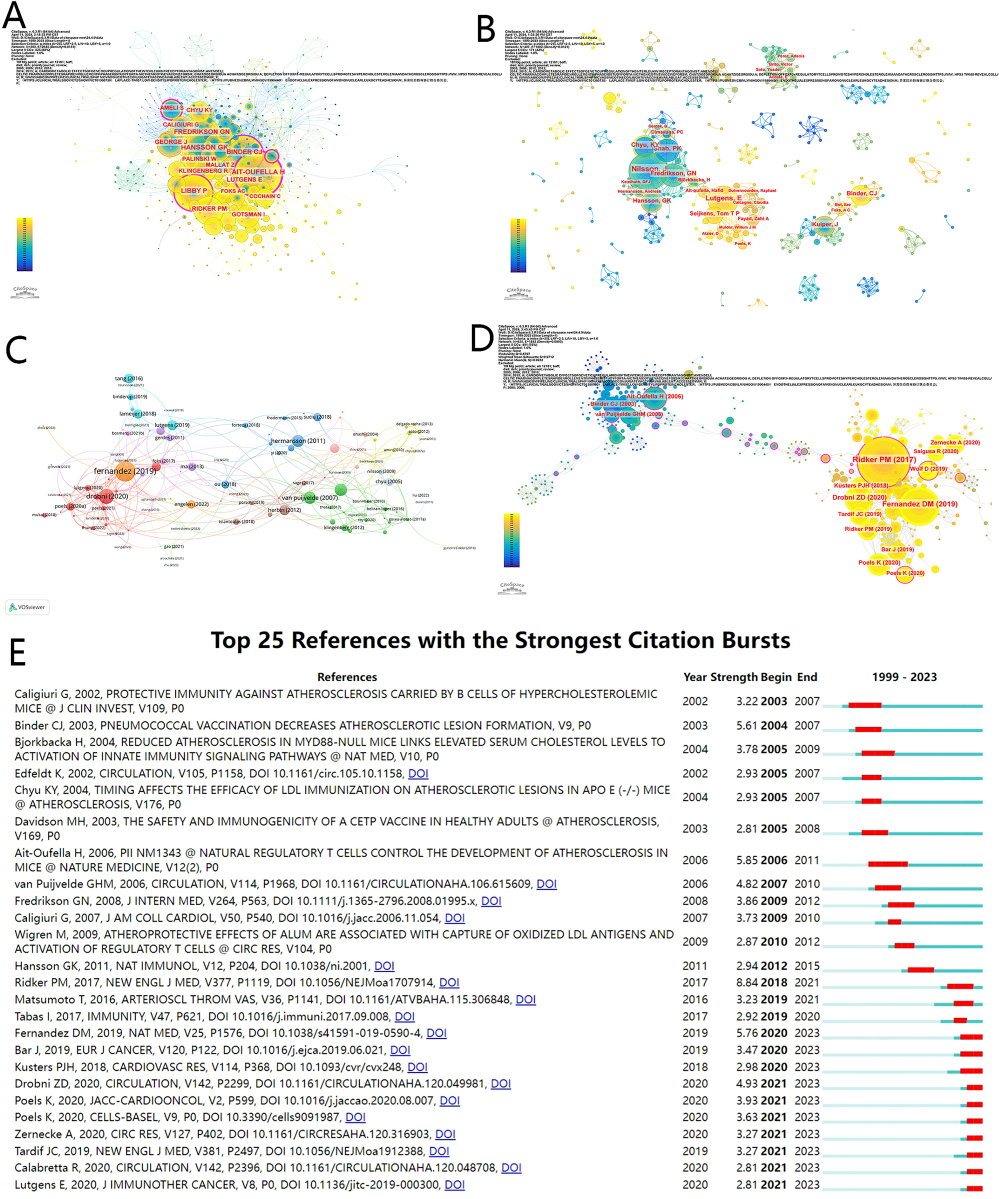
**(A)** Cocited author co-occurrence map in immunotherapy of atherosclerosis. **(B)** Author Co-occurrence map in immunotherapy of atherosclerosis. **(C)** Citations between documents **(D)** Co-cited reference in immunotherapy of atherosclerosis **(E)** Top 25 references with the citation burst. Year means the time one reference is published. Strength indicates the extent of the burst. Begin and end represent the burst time interval. The diagram on the right: light blue means times the article unpublished, and red period means burst time.

According to the author analysis, a total of 963 authors were identified by VOSviewer. Of these, Jan Nilsson has the most outputs (13 publications), followed by Esther Lutgens (10 publications), Gunilla N Fredrikson (8 publications), Prediman K Shah (8 publications), and Kuang-Yuh Chyu (8 publications) (**Supplementary Table S2**). However, the number of publications is not a reliable indicator of an author’s status; the number of citations is more representative of status (34). Johan Kuiper is the most frequently cited author (499), followed by Tomas G Neilan (253) and Esther Lutgens (236) in the top 14. In **Figure 3A**, 593 cocited authors are intercepted, with a connection of 2645. Of these, 2 authors are cocited more than 50 times, and the top 5 cocited authors are Hafid Ait-Oufella (n=60), Göran K Hansson (n=56), Christoph J Binder (n=49), Peter Libby (n=48) and Gunilla N Fredrikson (n=46) (**Supplementary Table S2**).

The co-occurrence maps of authors and cocited authors are shown in (**Figures 3A, B**), respectively. In **Figure 3B**, the map has 407 network nodes and 1002 connections, with Jan Nilsson, Prediman K Shah, Gunilla N Fredrikson, Kuang-Yuh Chyu and Göran K Hansson closely interconnected. Moreover, Jan Nilsson, Esther Lutgens, Johan Kuiper, Christoph J Binder and Acosta E serve as key nodes within the major clusters. However, the clusters of authors appear to be dispersed, with no individual author exhibiting centrality exceeding 0.1 in the co-occurrence map. This suggests that more collaborations and interactions between authors in this field should be established.

### Citations

Reference citation analysis is an approach to evaluate the influence of publications and can also identify representative articles with a high number of citations. The number of times the 174 documents were cited was determined by VOSviewer, and the results are shown in **Figure 3C**; the top 10 more frequently cited documents are displayed in **Supplementary Table S3**. “Single-cell immune landscape of human atherosclerotic plaques”, authored by Fernandez DM, received the most citations (373), followed by the “Association Between Immune Checkpoint Inhibitors With Cardiovascular Events and Atherosclerotic Plaque”, authored by Drobni ZD (203).

**Supplementary Table S4** shows the top 12 cocited references in this field, providing detailed information, including title, primary author, year of publication and number of citations, which includes 9 articles, 2 reviews and 1 communication. “Anti-inflammatory Therapy with Canakinumab for Atherosclerotic Disease” (14), which was published in The New England Journal of Medicine and authored by Ridker PM, is the most frequently cited article, with 27 citations. The visualized network of cocited articles was created via CiteSpace (**Figure 3D**). All the cocited references roughly fell into two clusters by year of publication: blue and yellow. In the occurrence map, several articles have a centrality over 0.1, indicating influential and fundamental research. Burst analysis of the reference data is shown in **Figure 3E**; the earliest reference burst was “Protective immunity against atherosclerosis carried by B cells of hypercholesterolemic mice” (35), which occurred between 2003 and 2007 and has the longest duration of occurrence. However, “Natural regulatory T cells control the development of atherosclerosis in mice” (36) has the longest burst duration (y=5), and “Antiinflammatory Therapy with Canakinumab for Atherosclerotic Disease” (14) has the strongest burstiness (strength=8.84). In total, 10 documents burst in 2023, beginning in 2020 and ending in 2021.

### Keyword co-occurrence, burst, clusters and timeline view

**Figure 4:**
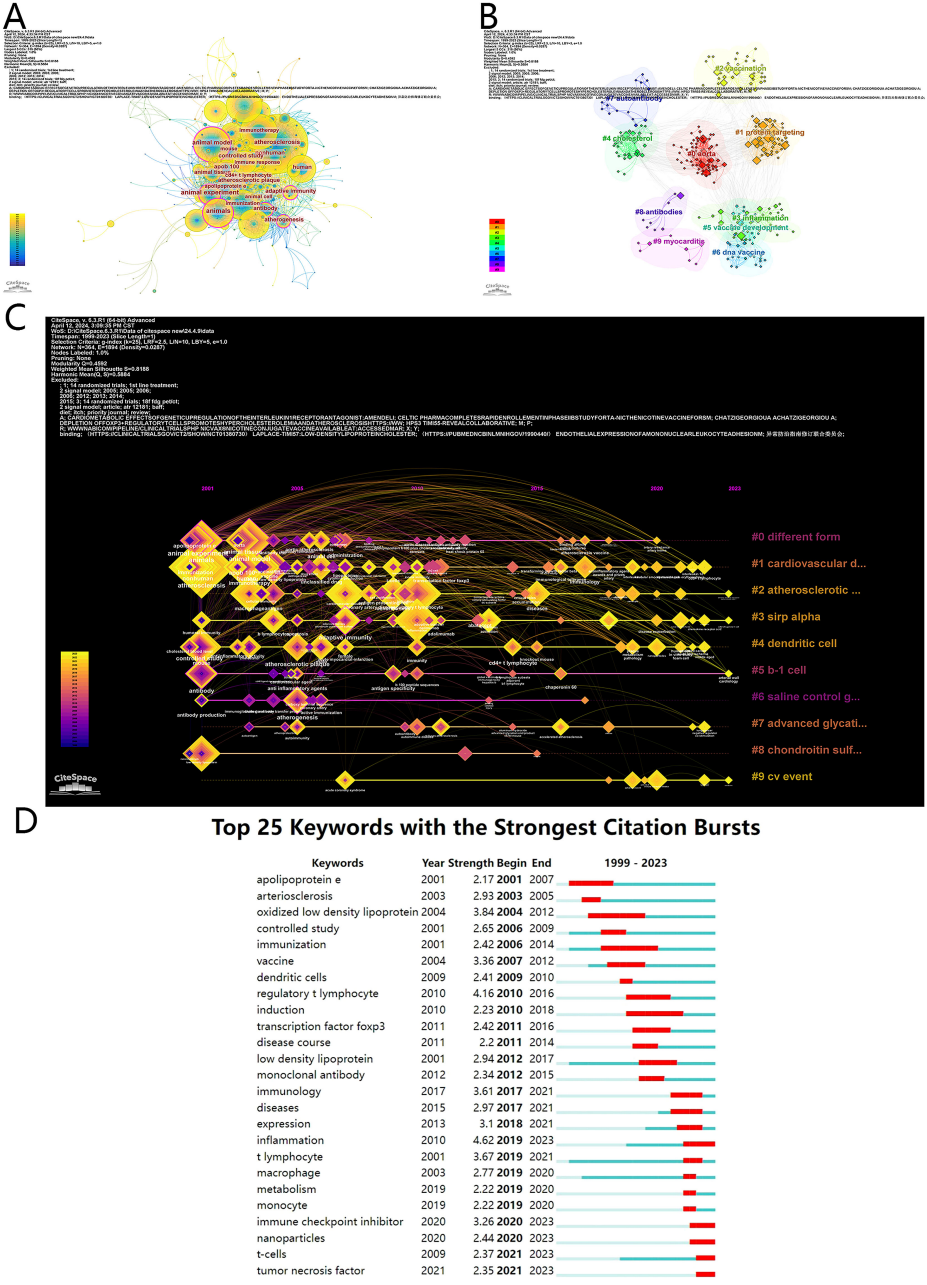
**(A)** Keyword Co-occurrence in immunotherapy of atherosclerosis. **(B)** Keyword cluster analysis. Different cluster represented as various clusters. **(C)** Keywords clustering timeline in immunotherapy of atherosclerosis. Keywords was displayed according to time and clusters, and each node represents a keyword. **(D)** Top 25 keywords with citation bursts.

Keywords play a core role in an article, and by analyzing keywords, we can explore hotspots in a research field (19). The keyword co-occurrence map was produced by CiteSpace, with 364 nodes and 1894 connections (**Figure 4A**). From the map, only 6 nodes surrounded by a purple circle (animals (0.18), animal experiment (0.15), antibody (0.14), atherogenesis (0.14), adaptive immunity (0.12), and animal model (0.11)) are included, and they have the role of lining other nodes (centrality>0.1) (**Figure 4A**). The top 20 keywords are displayed in **Supplementary Table S5**, and 10 words appeared more than 30 times. This table indicates that atherosclerosis is the most common content in this field (n=71), followed by nonhuman (n=49) and immunotherapy (n=43).

Clustering is an analysis method that classifies data by similarity (28). The clustering map of keywords has a Q-score of 0.4592 and an S-score of 0.8188. Information for the top 9 clusters is presented in **Supplementary Table S6**. Cluster 8 has the highest S value of 0.973 and includes myocarditis, immune checkpoint inhibitors, lung cancer, prevention, and immune mechanisms of cardiovascular disease, followed by Cluster 8 (0.968), which includes antibodies, oxidative stress, glycosaminoglycans, tolerization, and glycosaminoglycan. Cluster 7 (0.949) includes autoantibody, advanced glycation end products, hyperglycemia, autoimmunity, and autoantigens. **Figure 4B** shows the keyword clustering timeline map that combines the cluster and time span and intuitively presents the time progression of keywords (29). From 2001 to 2010, the main keywords were atherosclerosis, nonhuman, immunotherapy, animals, regulatory T cells and inflammation, whereas from 2010 to 2023, disease, immunology, and expression were the primary terms (**Figure 4C**).

Keyword burst analysis assesses hotspots in a period and demonstrates keyword trends (28, 37). The list of the 25 strongest keywords is shown in **Figure 4D**. Among the top 25 keywords, three keywords had the longest burst period of 8 years: oxidized low-density lipoprotein (2004–2012), immunization (2006–2014), and induction (2010–2018). Research hotspots include inflammation, immune checkpoint inhibitors, nanoparticles, T cells and tumor necrosis factor in 2023, and these topics may predict future research trends.

## Discussion

In this study, we conducted a bibliometric analysis of immunotherapy for atherosclerosis to gain insight into current research trends and hotspots in the field. The main findings were as follows: 1) annual publication growth is fluctuating; 2) the USA, China, and the Netherlands are leading countries, and the top institutions primarily in the Netherlands, Sweden, and the USA; 3) Jan Nilsson is the most prolific author; 4) key journals in this field include Arteriosclerosis Thrombosis and Vascular Biology, Frontiers in Cardiovascular Medicine, Circulation and Vaccine; 5) Ridker PM’s work on Canakinumab holds a prominent role; and 6) future research may focus on inflammation, nanoparticles, immune checkpoint inhibitors, T cells and tumor necrosis factors. This analysis not only addresses the previous gaps in understanding immunotherapy of atherosclerosis but also highlights potential collaborations and future research directions, guiding subsequent studies on the applications on immunotherapy in this field. The annual publication of studies highlights the papers and advances within this field. The initial research on the use of immunotherapy for atherosclerosis was published in 1999 and authored by L Capron, indicating that researchers recognized the importance of immunology and the use of immunotherapy for atherosclerosis in an early stage. The publication trends fluctuate overall, with significant turning points occurring in 2007, 2012, 2016 and most recently in 2021, indicating the significance of this topic as an area of long-term research interest. Although the number of publications has declined since 2022, future research still holds promising prospects. The annual growth pattern indicates promising prospects in this field.

An analysis of countries and institutions can identify highly influential regions and institutions, as well as their cooperation in the field of immunotherapy for atherosclerosis. The USA has a high degree of activity and significant authority in national collaboration in this field, with the most output, greatest centrality, and strongest link strength. Moreover, the papers published by the USA in the field have had a significant influence and made important contributions. Among the top ten cited articles from the USA, seven were published in journals with IFs over 10, highlighting their broad influence on academia and medicine. China ranks second in terms of the number of publications; however, the degree of centrality and number of citations are relatively low. This is partly because of the high self-citation rate because even though the number of publications accounts for 1.6% of the total number of publications worldwide, the self-citation rate is as high as 50%, indicating low international recognition; in addition, some papers are published for the sake of quantity, resulting in a large number of research results but not a strong impact (38). According to the analysis of institutions, eight institutions are located in European countries, and 2 in America. Moreover, America has the highest number of publications, showing that it has a wide range of institutions where this topic is studied. The diversity observed among countries and regions significantly contributes to advances in this field. Promoting interactions between different regions can reduce the obstacles to developing atherosclerosis immunotherapy. Among the top 10 institutions, the University of Amsterdam plays a crucial role by focusing on immune checkpoint inhibitors and their adverse effects on atherosclerosis, particularly in how these may promote inflammation. Its contributions also extend to the development of nano immunotherapy strategies (39–41). Lund University specializes in studying of the effects of apolipoprotein B100 on regulatory T cells and has developed various innovative immunization strategies, offering new therapeutic options for future clinical applications (42, 43). Harvard University stands out for its research on the development and application of nanoparticles specifically for atherosclerosis, bridging the gap between nanotechnology and immunotherapy (44, 45). The collaboration among these institutions is significant for advancing immunotherapy in atherosclerosis. Such interdisciplinary collaboration not only accelerates research but also paves the way for more effective treatments.

The bibliometric analysis of authors in the field of immunotherapy for atherosclerosis reveals a complex network of collaborations and influential researchers. Notably, Jan Nilsson and Esther Lutgens emerge as the top authors with the highest publication counts, whereas Hafid Ait-Oufella, Göran K Hansson, and Christoph J Binder are the most frequently cocited authors. Nilsson J, from the Department of Clinical Sciences, Malmö University Hospital, made a major contribution to understanding the relationship of regulatory T cells and apolipoprotein B with AS immunotherapy (46, 47). His work has provided critical insights into the combination of metabolism and immunity, promoting the development of effective therapeutic strategies. Lutgen E’s research has focused mainly on immune checkpoints (48, 49), and his research revealed that cancer patients receiving ICIs have an increased risk of AS, emphasizing the need to carefully manage these patients (50). This highlights the importance of his work in understanding the unintended consequences of immunotherapy, thereby guiding clinical practices. Furthermore, there are notable collaborations among these top 10 authors. Nilsson J, Shah PK, Chyu KY, and Hansson GK have engaged in various degrees of cooperation, indicating the importance of interdisciplinary research in this field (32). In addition to the number of publications, the number of citations reflects the impact and relevance of an author’s research on the scientific community, and a high citation count indicates that an author’s work has been widely validated. Kuiper J has the highest citation count, suggesting that their findings have significantly influenced subsequent research and clinical applications. The cocitation analysis reveals that Ait-oufella H, Hansson GK, and Libby P are the most frequently cocited authors. In addition, some of these highly productive authors are affiliated with the top 10 institutions in this field. For example, Nilsson is a researcher at Lund University, whereas Lutgens E is a researcher at the University of Munich. The bibliometric analysis of authorship identifies areas of potential collaboration in the field of immunotherapy for atherosclerosis. Future research should focus on integrating the expertise of top authors and institutions to address these gaps and find innovations in this field.

Journal usage in the field of immunotherapy for arteriosclerosis was evaluated, with the aim of providing publication recommendations for researchers. Most of the top 11 journals were professional journals from the cardiovascular and immune-related fields rather than comprehensive journals. This suggests that within this particular field, specialized journals tend to have a greater impact than comprehensive journals (34). Among the top 11 journals, 4 have an IF over 10, whereas nine have an IF over 5. Arteriosclerosis Thrombosis and Vascular Biology, Frontiers in Cardiovascular Medicine and Circulation are three most prominent journals with the highest number of articles on immunotherapy for atherosclerosis (42, 43, 51–56). Moreover, studies published in Molecular, Biology, and Genetics have been frequently cited by studies published in Molecular, Biology, and Immunology and Medicine, Medical, and Clinical, indicating a strong link between biogenetics and immunology in this field. In summary, the results of this study can help researchers choose appropriate journals and assist them in research achievements.

The analysis of citations can explore the implications of references for understanding the influence of publications and research trends in the field of atherosclerosis. Our findings highlight the significance of “Single-cell immune landscape of human atherosclerotic plaques” (57). This research analyzed the single-cell proteome and transcriptome to identify differences in immune cells between carotid artery plaques and blood from patients with and without recent strokes, providing more accurate information on alterations in immune cells after atherosclerosis (57). The research focus has evolved from an initial emphasis on animal experiments in earlier studies (36, 58) to a more clinical orientation in recent research (11, 14), suggesting a growing interest in translational investigations in humans. The reference burst map displays the most cited references during a specific period, indicating the research interests of those times. Notably, the earliest published and earliest burst article was “Protective immunity against atherosclerosis carried by B cells of hypercholesterolemic mice”, authored by Giuseppina Caligiuri in 2002, which demonstrated the role of B cells in the spleen in atherosclerosis (35). The results also identify the study with the longest burst time, “Natural regulatory T cells control the development of atherosclerosis in mice” (36), and the reference with the highest burst strength, “Anti-inflammatory Therapy with Canakinumab for Atherosclerotic Disease” (14). Both have high IFs (82.9 and 158.5, respectively) and have contributed significantly to this field. Recently bursting publications with high impact factors has focused on the effects of low-dose colchicine and immune checkpoint inhibitors on patients with cardiovascular disease (16, 59–61). This trend suggests a growing recognition of the importance of more precise medicine in treatment. In conclusion, our citation analysis provides a comprehensive understanding of atherosclerosis research, highlighting influential articles, research trends, and shifting orientations.

In the field of atherosclerosis, keyword analysis provides valuable insights into the dynamic evolution of research interests and trends. The results revealed that the five most frequently occurring keywords were “atherosclerosis”, “nonhuman”, “immunotherapy”, “animal model” and “regulatory T lymphocyte”, indicating that the literature in this field has focused primarily on investigating the effects of immunotherapy on atherosclerosis through animal experiments. This demonstrates that preclinical studies are a solid foundation for research, which is essential for understanding the underlying mechanisms of atherosclerosis and the potential for immunotherapy intervention. Notably, cluster analysis highlights the significance of terms such as “adaptive immunity”, “protein targeting”, “vaccination”, “inflammation”, “cholesterol”, “apob 100”, “DNA vaccine”, “autoantibody”, “antibody” and “myocarditis” in shaping the knowledge structure of the field. These keywords demonstrate the multifaceted nature of atherosclerosis research, including adaptive immune responses, protein-specific targets, vaccination strategies, and the role of inflammation and lipids in the pathogenesis of atherosclerosis. According to the timeline view, research in this field began with animal experiments targeting potential immune targets, such as low-density lipoprotein and active T lymphocytes in 2001. This early stage uses animal experiments to focus on potential targets, laying the foundation for explorations. From 2005 to 2015, significant advances were made, with the discovery of novel molecules and cell targets such as apolipoprotein B, antigen-presenting cells, dendritic cells, and the transcription factor Focp3. A series of new lipid and immune targets have been gradually discovered in this stage, revealing the diversity and complexity of the AS process. Since 2020, emerging keywords such as “nanoparticles”, “immune checkpoint inhibitors”, and “adverse effects” have shifted towards more sophisticated and targeted approaches. For example, a recent study used a nanoparticle assembled from a low molecular weight heparin-lipoic acid conjugate and curcumin to deliver reactive oxygen species to prevent the migration of monocytes to endothelial cells. This research achieved precision delivery, suppression of ROS, and effective anti-atherosclerosis functions, as confirmed by imaging, pathology, and serum analysis (62). Additionally, the utilization of immune checkpoint inhibitors has been shown to aggravate atherosclerosis due to their facilitation for T cell activation (63). Preclinical data indicate that abnormal interactions between PD-1/PD-L1 enhance the infiltration of macrophages and T cells into atherosclerotic plaques, highlighting the adverse effects that 3promote the advancement of atherosclerosis (64). However, some researchers highlight the potential of targeting immune checkpoints, especially in regulating inflammation. For instance, inhibiting the CD40L-CD40 pathway can prevent inflammatory macrophage recruitment, leading to the development of precise therapies that modulate inflammation and improve outcomes in atherosclerosis (65). Although research on immune checkpoints in atherosclerosis is still in its early stages, scientists propose that inhibiting co-stimulatory immune checkpoints or activating co-inhibitory immune checkpoints could effectively reduce inflammation and promote immune tolerance, which may help inhibit the development and progression of atherosclerosis (66).

From the table of keyword bursts, it was observed that keywords related to lipids emerged initially. Over time, there was a noticeable increase in immune-related keywords, highlighting the discovery of the effect of immunity to AS. Furthermore, among the top 25 keywords with the strongest citation bursts, “inflammation” and “regulatory T lymphocytes” emerged as particularly notable. The longest-lasting bursts of 8 years were observed for “oxidized low-density lipoprotein”, “immunization” and “induction”, whereas “apolipoprotein E” and “regulatory T lymphocyte” had the second longest-lasting burst. This indicates a sustained and intense interest in these areas over extended periods. In addition, an analysis of recent keyword bursts shows that inflammation continues to be a focal point of interest, indicating a consistent direction for future research. Furthermore, advancements in immune checkpoint modulators and nanotechnology are expected to progress, thereby facilitating the development of immunotherapy for atherosclerosis. These keywords not only highlight research hotspots during certain periods but also reflect current research progress, serving as principal nodes for enhancing understanding within this field. The association of these keywords with immunotherapy and atherosclerosis highlights the complexity of the research landscape in this field and the diverse range of approaches being explored to develop effective immunotherapies for atherosclerosis.

Atherosclerosis is a complex disease characterized by lipid accumulation and inflammation, presenting multiple immunotherapy targets. Approaches to immunotherapy for AS focus on several key mechanisms. One strategy involves immunization with oxidized LDL (ox-LDL) or its modified forms. This method directly targets lipid accumulation and induces an immune response to clear oxidized lipids from plaques (67, 68). Another approach modulates inflammation by targeting components involved in the immune response. These components include pro-inflammatory molecules like VEGFR2 (55), enhancing regulatory T cells with agents such as HCW9302 (69), modulating macrophage function (70), and targeting cytokines (71). In addition, immune checkpoints provide a broader modulation of immune response by regulating T cell activation and tolerance through targets like CD28-CD80/86, PD-1, and CTLA-4 (39, 49, 72). Nanotechnology also presents a promising approach by offering targeted delivery of immunomodulatory agents, reshaping the plaque microenvironment, and improving innate immunity (73–76). Moreover, additional molecular targets, including heat shock proteins, apolipoprotein B100, and collagen, further expand the scope of immunotherapeutic strategies. The pathogenesis of atherosclerosis reveals numerous potential targets for immunotherapy. Therefore, future studies could explore combination therapies to optimize delivery methods and enhance effectiveness. The current review represents the first bibliometric analysis of the use of immunotherapy for atherosclerosis, providing a quantitative assessment in this area. However, this study still has some limitations. The article selection was conducted manually, which may have resulted in missed or incorrect selections. This exclusion of relevant studies could lead to an incomplete representation of the current research landscape, potentially making the general trends misleading. Additionally, the literature was sourced from three databases and combined using CiteSpace, which may introduce inaccuracies regarding the information in each publication. This condition could lead to missing or duplicate data, affecting the accuracy of the outcomes. Furthermore, although these three datasets are commonly used in medical research, some publications from other datasets may have been overlooked. Moreover, our research focused specifically on immunotherapy for atherosclerosis, which excluded relevant contributions from studies on broader topics that could provide valuable insights. This narrow focus further restricts the scope of the analysis. Despite variations in data quality, this bibliometric analysis identifies key areas of interest and future trends within this field. To address these limitations, involving multiple researchers in the article selection process could enhance accuracy and reduce bias. Utilizing ,alternative merging software could also improve data integration. Expanding the included databases would ensure a more comprehensive literature review, and broadening the scope of the topic could enhance article filtering.

## Conclusion

Researches related to the use of immunotherapy for atherosclerosis have grown in a fluctuating manner between 1999 and 2023. However, collaboration between authors must be further improved, as fragmented clusters of authors were observed. To improve research productivity and innovation, establishing more robust collaborations could be beneficial. For instance, the University of Amsterdam primarily focuses on immune checkpoints, while Lund University specializes in apolipoproteins. A partnership between these institutions could yield significant insights into immunology and lipid metabolism in atherosclerosis. Besides, future studies may focus on specific areas, including the role of inflammation in atherosclerosis, the application of nanoparticles for target delivery of immunotherapies, and an exploration of adverse events associated with immune checkpoint inhibitors. In addition, investigating the mechanism behind T cells and tumor necrosis factor could also provide valuable insights.

These findings illuminate the current state of research in immunotherapy for atherosclerosis and have the potential to enhance clinical practice. Research from influential institutions can pave the way for innovative therapies based on novel hotspots identified, such as using immune checkpoint modulators and nanoparticle delivery systems, leading to the development of personalized treatments. Leading countries like the USA, are likely to influence global treatment strategies by high-impact publications, offering clinicians new practices that can improve patient outcomes. In the long term, the successful implementation of immunotherapy strategies could not only enhance the management of atherosclerosis but also contribute to the promotion of personalized medicine in cardiovascular care.

## Data Availability

The original contributions presented in the study are included in the article/**Supplementary Material**. Further inquiries can be directed to the corresponding author.
