# Supplementary material for: A bibliometric analysis of immunotherapy for atherosclerosis: trends and hotspots prediction
Source: Front Immunol. 2024 Nov 19;15:1493250. doi: 10.3389/fimmu.2024.1493250 (PMC11611808; doi:10.3389/fimmu.2024.1493250)
Supplement: Supplementary file 1 [file DataSheet1.docx]

**A Bibliometric Analysis of Immunotherapy for Atherosclerosis: Trends and Hotspots Prediction**

Jing-Hui Wang^1,2†^, Guan-Rui Pan^1,2†^, Long Jiang^1^*

Correspondence: Long Jiang* [skyiadx@hotmail.com](mailto:skyiadx@hotmail.com)

†These authors share first author

**Supplementary Data**

**Table S1 Impact Factor of journals (2023)**

**Table S2 Top 14 authors & co-cited authors**

**Table S3 Top 10 cited documents**

**Table S4 Top 12 co-cited references**

**Table S5 Top 20 keywords**

**Table S6 Information for 9 cluster**

**Fig. S1 The process of literature screening**

**Table S1 Impact Factor of journals (2023)**

| Rank | Journal | Count | IF |
| --- | --- | --- | --- |
| 1. | Arteriosclerosis thrombosis and vascular biology | 14 | 7.4 |
| 2. | Frontiers in cardiovascular medicine | 6 | 2.8 |
| 3. | Circulation | 5 | 35.5 |
| 4. | Vaccine | 5 | 4.5 |
| 5. | CARDIOVASCULAR RESEAECH | 4 | 10.9(2022) |
| 6. | Frontiers in immunology | 4 | 5.7 |
| 7. | JACC: CardioOncology | 4 | 12 |
| 8. | American journal of physiology | 3 | 4.1 |
| 9. | Atherosclerosis | 3 | 4.9 |
| 10. | European heart journal | 3 | 37.6 |
| 11. | Journal of the American college of cardiology (JACC) | 3 | 21.7 |
| 12. | Biomaterials Science | 2 | 5.8 |
| 13. | British Journal of Pharmacology | 2 | 6.8 |
| 14. | Cells | 2 | 5.1 |
| 15. | Current pharmacology design | 2 | 3.1(2022) |
| 16. | Frontiers in Pharmacology | 2 | 4.4 |
| 17. | immunobiology | 2 | 2.5 |
| 18. | INTERNATIONAL IMMUNOPHARMACOLOGY | 2 | 4.8 |
| 19. | Journal for immunotherapy of cancer | 2 | 10.3 |
| 20. | Journal of immunology | 2 | 3.6 |
| 21. | Journal of Shanghai Jiaotong University (Medical Science) | 2 | / |
| 22. | MOLECULAR BIOTECHNOLOGY | 2 | 2.4 |
| 23. | Seminars in Immunopathology | 2 | 7.9 |
| 24. | Vascular pharmacology | 2 | 3.5 |
| 25. | ACS Nano | 1 | 15.8 |
| 26. | ACTA PHARMACOLOGICA SINICA | 1 | 6.9 |
| 27. | ADVANCED FUNCTIONAL MATERIALS | 1 | 18.5 |
| 28. | Advanced Healthcare Materials | 1 | 10.0 |
| 29. | Aging-US | 1 | 3.9 |
| 30. | AMERICAN HEART JOURNAL | 1 | 3.7 |
| 31. | Archives of medical research | 1 | 4.7 |
| 32. | ANNALS OF MEDICINE | 1 | 4.9 |
| 33. | APL Bioengineering | 1 | 6.6 |
| 34. | Archives of Medical Science | 1 | 3.0 |
| 35. | AUTOIMMUNITY REVIEWS | 1 | 9.2 |
| 36. | BIOCHEMICAL AND BIOPHYSICAL RESEARCH COMMUNICATIONS | 1 | 2.5 |
| 37. | Biology-Basel | 1 | 3.6 |
| 38. | BIOMEDICINE and PHARMACOTHERAPY | 1 | 6.9 |
| 39. | Biomedicines | 1 | 3.9 |
| 40. | BMC BIOLOGY | 1 | 4.4 |
| 41. | BMC Medical Genomics | 1 | 2.1 |
| 42. | Cancers | 1 | 4.5 |
| 43. | Cardio-Oncology | 1 | 3.2 |
| 44. | CARDIOVASCULAR PATHOLOGY | 1 | 2.3 |
| 45. | Cardiovascular Therapeutics | 1 | 3.4 |
| 46. | CELL STRESS and CHAPERONES | 1 | 3.3 |
| 47. | Cellular and Molecular Immunology | 1 | 24.1(2022) |
| 48. | CIRCULATION RESEARCH | 1 | 16.5 |
| 49. | CLINICA CHIMICA ACTA | 1 | 3.2 |
| 50. | CLINICAL and EXPERIMENTAL IMMUNOLOGY | 1 | 3.4 |
| 51. | CLINICAL IMMUNOLOGY | 1 | 4.5 |
| 52. | Current Atherosclerosis Reports | 1 | 5.7 |
| 53. | Current Drug Therapy | 1 | 0.3 |
| 54. | CURRENT OPINION IN LIPIDOLOGY | 1 | 3.8 |
| 55. | Current Opinion in Molecular Therapeutics | 1 | 3.416 (2012) |
| 56. | Current Opinion in Organ Transplantation | 1 | 1.8 |
| 57. | CURRENT PROBLEMS IN CARDIOLOGY | 1 | 3.0 |
| 58. | Endocrine Metabolic and Immune Disorders-Drug Targets | 1 | 1.9(2022) |
| 59. | EUROPEAN JOURNAL OF VASCULAR and ENDOVASCULAR SURGERY | 1 | 5.7 |
| 60. | EXPERT OPINION ON BIOLOGICAL THERAPY | 1 | 3.6 |
| 61. | EXPERT OPINION ON PHARMACOTHERAPY | 1 | 2.5 |
| 62. | EXPERT OPINION ON THERAPEUTIC PATENTS | 1 | 5.4 |
| 63. | Expert Review of Clinical Immunology | 1 | 3.9 |
| 64. | Expert Review of Vaccines | 1 | 5.5 |
| 65. | FASEB JOURNAL | 1 | 4.4 |
| 66. | FREE RADICAL BIOLOGY and MEDICINE | 1 | 7.1 |
| 67. | FREE RADICAL RESEARCH | 1 | 3.6 |
| 68. | Frontiers in Bioengineering and Biotechnology | 1 | 4.3 |
| 69. | Hamostaseologie | 1 | 2.7 |
| 70. | Handbook of Experimental Pharmacology | 1 | / |
| 71. | IMMUNOLOGY LETTERS | 1 | 3.3 |
| 72. | Immunotherapy | 1 | 2.7 |
| 73. | INFECTION and IMMUNITY | 1 | 2.9 |
| 74. | INFLAMMATION RESEARCH | 1 | 4.8 |
| 75. | International Heart Journal | 1 | 1.2 |
| 76. | INTERNATIONAL JOURNAL OF CARDIOLOGY | 1 | 3.2 |
| 77. | INTERNATIONAL JOURNAL OF MOLECULAR SCIENCES | 1 | 4.9 |
| 78. | INTERNATIONAL REVIEWS OF IMMUNOLOGY | 1 | 4.3 |
| 79. | JACC-Basic to Translational Science | 1 | 8.4 |
| 80. | JAPANESE JOURNAL OF CLINICAL ONCOLOGY | 1 | 1.9 |
| 81. | JCI Insight | 1 | 6.3 |
| 82. | Journal of Atherosclerosis and Thrombosis | 1 | 3.0 |
| 83. | JOURNAL OF BIOLOGICAL CHEMISTRY | 1 | 4.0 |
| 84. | JOURNAL OF BIOMATERIALS APPLICATIONS | 1 | 2.3 |
| 85. | Journal of Cardiovascular Translational Research | 1 | 2.4 |
| 86. | Journal of China Pharmaceutical University | 1 | / |
| 87. | JOURNAL OF CONTROLLED RELEASE | 1 | 10.5 |
| 88. | JOURNAL OF DENTAL RESEARCH | 1 | 5.7 |
| 89. | JOURNAL OF MOLECULAR and CELLULAR CARDIOLOGY | 1 | 4.9 |
| 90. | JOURNAL OF THE AMERICAN SOCIETY OF ECHOCARDIOGRAPHY | 1 | 5.4 |
| 91. | Lipids in Health and Disease | 1 | 3.9 |
| 92. | MEDICAL HYPOTHESES | 1 | 2.1 |
| 93. | MEDICAL SCIENCE MONITOR | 1 | 2.2 |
| 94. | Medicine | 1 | 1.3 |
| 95. | METABOLISM-CLINICAL and EXPERIMENTAL | 1 | 10.8 |
| 96. | Nano Today | 1 | 13.2 |
| 97. | Nature Biomedical Engineering | 1 | 26.8 |
| 98. | Nature Clinical Practice Cardiovascular Medicine | 1 | 7.043 (2011) |
| 99. | Nature medicine | 1 | 58.7 |
| 100. | Nature Reviews Cardiology | 1 | 41.7 |
| 101. | PHARMACOLOGY and THERAPEUTICS | 1 | 12.0 |
| 102. | PLANT CELL REPORTS | 1 | 5.3 |
| 103. | PROCEEDINGS OF THE NATIONAL ACADEMY OF SCIENCES OF THE UNITED STATES OF AMERICA | 1 | 9.4 |
| 104. | QJM-AN INTERNATIONAL JOURNAL OF MEDICINE | 1 | 7.3 |
| 105. | RUSSIAN CHEMICAL BULLETIN | 1 | 1.7 |
| 106. | Science Translational Medicine | 1 | 15.8 |
| 107. | Scientific Reports | 1 | 3.8 |
| 108. | Theranostics | 1 | 12.4 |
| 109. | Therapeutic Advances in Medical Oncology | 1 | 4.3 |
| 110. | THROMBOSIS and HAEMOSTASIS | 1 | 5.0 |
| 111. | TRANSPLANTATION PROCEEDINGS | 1 | 0.8 |
| 112. | TRENDS IN CARDIOVASCULAR MEDICINE | 1 | 7.3 |
| 113. | VacciMonitor | 1 | / |
| 114 | Cardiovascular Diabetology | 1 | 8.5 |
| 115 | Cardiology in Review | 1 | 2.0 |
| 116 | CARDIOVASCULAR DRUGS and THERAPY | 1 | 3.1 |

**Table S2 Top 14 authors & co-cited authors**

| Rank | Author | Count | Citations | Centrality | Co-cited author | Co-citation |
| --- | --- | --- | --- | --- | --- | --- |
| 1 | Jan Nilsson | 13 | 200 | 0.01 | Hafid Ait-Oufella | 60 |
| 2 | Esther Lutgens | 10 | 236 | 0.04 | Göran K Hansson | 56 |
| 3 | Prediman K Shah | 8 | 62 | 0.00 | Christoph J Binder | 49 |
| 4 | Gunilla N Fredrikson | 8 | 160 | 0.05 | Peter Libby | 48 |
| 5 | Kuang-Yuh Chyu | 8 | 64 | 0.00 | Gunilla N Fredrikson, | 46 |
| 6 | Johan Kuiper | 7 | 499 | 0.01 | Paul M Ridker | 38 |
| 7 | Christoph J Binder | 7 | 102 | 0.01 | Jacob George | 35 |
| 8 | Göran K Hansson | 7 | 202 | 0.02 | Esther Lutgens | 34 |
| 9 | Tom T P Seijkens | 6 | 139 | 0.00 | Roland Klingenberg | 32 |
| 10 | Paul C Dimayuga | 4 | 62 | 0.01 | Wulf Palinski | 32 |
| 11 | Hafid Ait-Oufella | 4 | 102 | 0.04 | Israel Gotsman | 30 |
| 12 | Zahi A Fayad | 4 | 149 | 0.00 | Kuang-Yuh Chyu | 28 |
| 13 | Tomas G Neilan | 4 | 253 | 0.00 | Ziad Mallat | 28 |
| 14 | Harry Björkbacka | 4 | 3 | 0.00 | Giuseppina Caligiuri | 27 |

**Table S3 Top 10 cited documents**

| Rank | Title | First author | Year | Citations |
| --- | --- | --- | --- | --- |
| 1 | Single-cell immune landscape of human atherosclerotic plaques | Fernadez DM | 2019 | 373 |
| 2 | Association Between Immune Checkpoint Inhibitors With Cardiovascular Events and Atherosclerotic Plaque | Drobin ZD | 2020 | 203 |
| 3 | Induction of oral tolerance to HSP60 or an HSP60-peptide activates T cell regulation and reduces atherosclerosis | van Puijvelde HM | 2007 | 142 |
| 4 | Induction of oral tolerance to oxidized low-density lipoprotein ameliorates atherosclerosis | van Puijvelde HM | 2006 | 141 |
| 5 | Immunotherapy With Tolerogenic Apolipoprotein B-100-Loaded Dendritic Cells Attenuates Atherosclerosis in Hypercholesterolemic Mice | Hermansson A | 2011 | 135 |
| 6 | Regulatory T-Cell Response to Apolipoprotein B100-Derived Peptides Reduces the Development and Progression of Atherosclerosis in Mice | Herbin O | 2012 | 98 |
| 7 | Immunotherapy for cardiovascular disease | Lutgens E | 2019 | 90 |
| 8 | Immune cell screening of a nanoparticle library improves atherosclerosis therapy | Tang J | 2016 | 84 |
| 9 | Efficacy and safety assessment of a TRAF6-targeted nanoimmunotherapy in atherosclerotic mice and non-human primates | Lameijer M | 2018 | 81 |
| 10 | Vaccination using oxidized low-density lipoprotein-pulsed dendritic cells reduces atherosclerosis in LDL receptor-deficient mice | Habets KLL | 2010 | 80 |

**Table S4 Top 12 co-cited references**

| Rank | Title | First author | Year | Type | Journal | Citation |
| --- | --- | --- | --- | --- | --- | --- |
| 1 | Antiinflammatory Therapy with Canakinumab for Atherosclerotic Disease | Ridker PM | 2017 | Article | NEW ENGL J MED | 27 |
| 2 | Single-cell immune landscape of human atherosclerotic plaques | Fernandez DM | 2019 | Article | NAT MED | 21 |
| 3 | Association Between Immune Checkpoint Inhibitors With Cardiovascular Events and Atherosclerotic Plaque | Drobni ZD | 2020 | Article | CIRCULATION | 15 |
| 4 | Natural regulatory T cells control the development of atherosclerosis in mice | Ait-Oufella H | 2006 | Communication | NAT MED | 12 |
| 5 | Immune Checkpoint Inhibitor Therapy Aggravates T Cell-Driven Plaque Inflammation in Atherosclerosis | Poels K | 2020 | Article | JACC-CARDIOONCOL | 12 |
| 6 | Exploring immune checkpoints as potential therapeutic targets in atherosclerosis | Kusters PJH | 2018 | Article | CARDIOVASC RES | 11 |
| 7 | Acute vascular events as a possibly related adverse event of immunotherapy: a single-institute retrospective study | Bar J | 2019 | Article | EUR J CANCER | 10 |
| 8 | Pneumococcal vaccination decreases atherosclerotic lesion formation: molecular mimicry between Streptococcus pneumoniae and oxidized LDL | Binder CJ | 2003 | Article | Nat Med | 10 |
| 9 | Low-Dose Methotrexate for the Prevention of Atherosclerotic Events | Ridker PM | 2019 | Article | NEW ENGL J MED | 10 |
| 10 | Efficacy and Safety of Low-Dose Colchicine after Myocardial Infarction | Tardif JC | 2019 | Article | NEW ENGL J MED | 10 |
| 11 | Immunity and Inflammation in Atherosclerosis | Wolf D | 2019 | Review | CIRC RES | 10 |
| 12 | Meta-Analysis of Leukocyte Diversity in Atherosclerotic Mouse Aortas | Zernecke A | 2020 | Review | CIRC RES | 10 |

| Rank | Keyword | Count | Centrality | Rank | Keyword | Count | Centrality |
| --- | --- | --- | --- | --- | --- | --- | --- |
| 1 | Atherosclerosis | 71 | 0.09 | 11 | T lymphocyte | 28 | 0.01 |
| 2 | Nonhuman | 49 | 0.04 | 12 | Low density lipoprotein | 27 | 0.04 |
| 3 | Immunotherapy | 43 | 0.01 | 13 | Cardiovascular disease | 27 | 0.03 |
| 4 | Animal model | 41 | 0.11 | 14 | Control study | 26 | 0.04 |
| 5 | Regulatory T lymphocyte | 41 | 0.04 | 15 | Macrophage | 25 | 0.02 |
| 6 | Immunization | 36 | 0.04 | 16 | Dendritic cell | 25 | 0.07 |
| 7 | Inflammation | 35 | 0.00 | 17 | Animal experiment | 24 | 0.15 |
| 8 | Animals | 34 | 0.18 | 18 | Apob 100 | 20 | 0.08 |
| 9 | Human | 33 | 0.05 | 19 | Oxidized low density lipoprotein | 20 | 0.01 |
| 10 | Mouse | 30 | 0.01 | 20 | Immune response | 19 | 0.01 |

**Table S5 Top 20 keywords**

**Table S6 Information for 9 cluster**

| Cluster ID | Size | Silhouette (S) | Mean year | Top terms |
| --- | --- | --- | --- | --- |
| #0 | 62 | 0.713 | 2007 | aorta; adaptive immunity; t lymphocyte; review; antibody specificity |
| #1 | 53 | 0.648 | 2009 | protein targeting; cholesterol; t lymphocyte receptor; apolipoprotein b100; vaccination |
| #2 | 39 | 0.946 | 2012 | vaccination; dendritic cells; mouse; mice; immunization |
| #3 | 38 | 0.858 | 2011 | inflammation; female; t cells; adaptive immunity; controlled study |
| #4 | 32 | 0.789 | 2013 | cholesterol; innate immunity; mouse; high density lipoprotein; supramolecular chemistry |
| #5 | 31 | 0.867 | 2010 | apob100; dexamethasone; vaccine development; atheroma; myocardial infarction |
| #6 | 17 | 0.936 | 2007 | dna vaccine; rabbit; hepatitis b core antigens; coronary artery; vaccines, dna |
| #7 | 16 | 0.949 | 2013 | autoantibody; advanced glycation end product; hyperglycemia; autoimmunity; autoantigen |
| #8 | 10 | 0.968 | 2011 | antibodies; oxidative stress; glycosaminoglycans; tolerization; glycosominoglycan |
| #9 | 8 | 0.973 | 2018 | myocarditis; immune checkpoint inhibitors; lung cancer; prevention; immune mechanisms of cardiovascular disease |


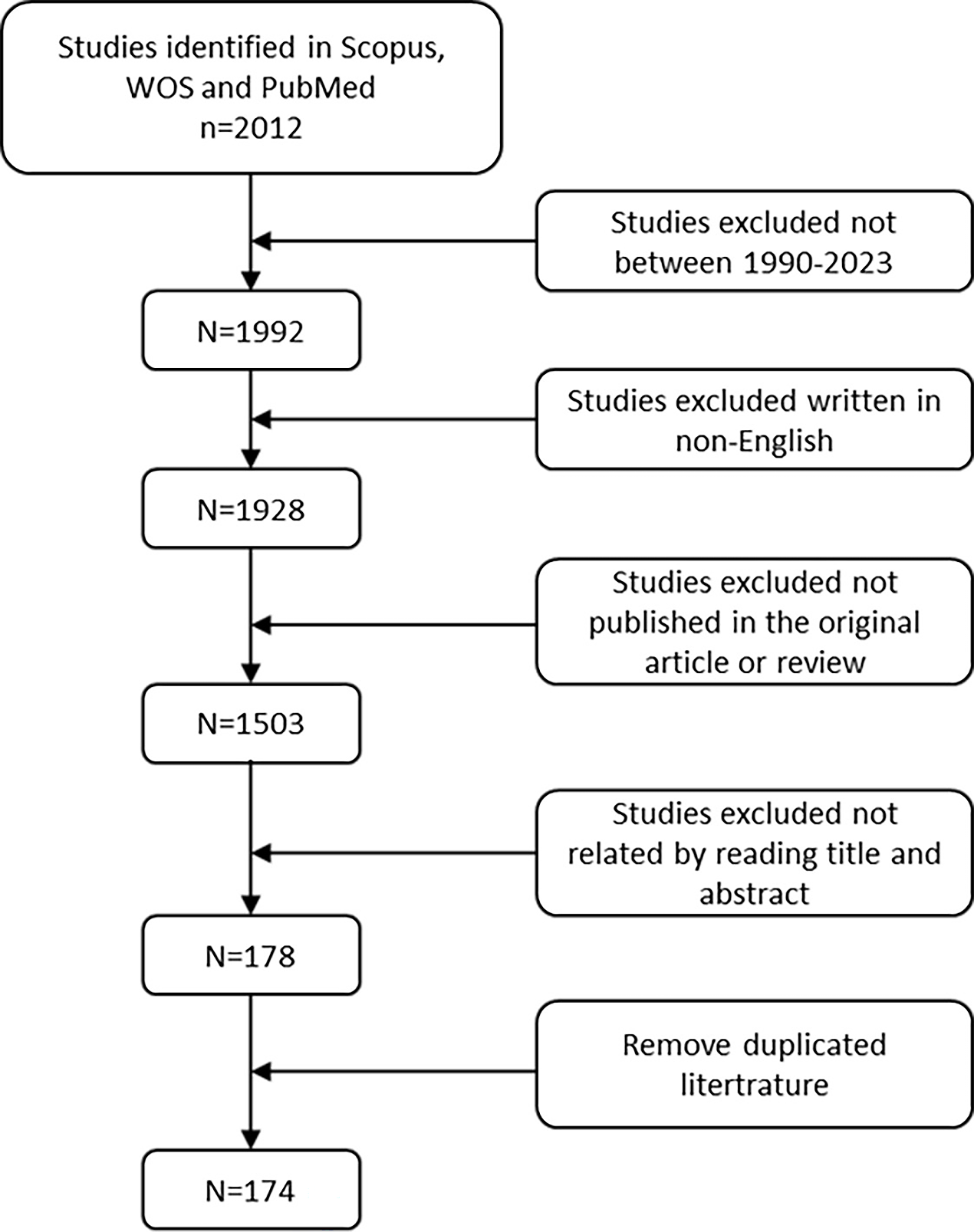


**Fig. S1 The process of literature screening.**
